# Correction: COVID-19 vaccine effectiveness among South Asians in Canada

**DOI:** 10.1371/journal.pgph.0004967

**Published:** 2025-07-22

**Authors:** Rahul Chanchlani, Baiju R. Shah, Shrikant I. Bangdiwala, Russell J. de Souza, Jin Luo, Shelly Bolotin, Dawn M. E. Bowdish, Dipika Desai, Karl Everett, Scott A. Lear, Mark Loeb, Zubin Punthakee, Diana Sherifali, Gita Wahi, Sonia S. Anand

The Acknowledgement section for this article is incorrect. The correct Acknowledgement section is as follows: Parts or whole of this material are based on data and/or information compiled and provided by Immigration, Refugees and Citizenship Canada (IRCC) current to May 2022. However, the analyses, conclusions, opinions and statements expressed in the material are those of the author(s), and not necessarily those of IRCC.
