# Supplementary material for: COVID-19 vaccine effectiveness among South Asians in Canada
Source: PLOS Glob Public Health. 2024 Aug 1;4(8):e0003490. doi: 10.1371/journal.pgph.0003490 (PMC11293718; doi:10.1371/journal.pgph.0003490)
Supplement: S3 Table — (DOCX) [file pgph.0003490.s003.docx]

**S3 Table: Baseline characteristics of the overall cohort stratified by covid-19 test results**

| **Characteristics** | **Value** | **People with negative covid-19 test and symptoms** | **People with positive covid-19 test and symptoms** | **Standardized difference** |
| --- | --- | --- | --- | --- |
|  |  | N=757,139 | N=126,016 |  |
|  |  |  |  |  |
| Sex | F | 442,520 (58.4%) | 65,968 (52.3%) | 0.12 |
|  | M | 314,619 (41.6%) | 60,048 (47.7%) | 0.12 |
|  |  |  |  |  |
| Age at index date | Mean ± SD | 44.08 ± 17.76 | 42.11 ± 16.72 | 0.11 |
|  | Median (IQR) | 41 (30-57) | 40 (28-54) | 0.1 |
|  |  |  |  |  |
| Age group | 18-29 | 185,744 (24.5%) | 35,791 (28.4%) | 0.09 |
|  | 30-39 | 173,645 (22.9%) | 26,981 (21.4%) | 0.04 |
|  | 40-49 | 127,884 (16.9%) | 22,541 (17.9%) | 0.03 |
|  | 50-59 | 111,808 (14.8%) | 20,392 (16.2%) | 0.04 |
|  | 60-69 | 83,376 (11.0%) | 11,815 (9.4%) | 0.05 |
|  | 70-79 | 44,800 (5.9%) | 5,266 (4.2%) | 0.08 |
|  | 80+ | 29,882 (3.9%) | 3,230 (2.6%) | 0.08 |
|  |  |  |  |  |
| Rural | missing | 1,994 (0.3%) | 341 (0.3%) | 0 |
|  | N | 673,079 (88.9%) | 119,262 (94.6%) | 0.21 |
|  | Y | 82,066 (10.8%) | 6,413 (5.1%) | 0.21 |
|  |  |  |  |  |
| Income Quintile | missing | 2,347 (0.3%) | 431 (0.3%) | 0.01 |
|  | 1 | 134,245 (17.7%) | 28,395 (22.5%) | 0.12 |
|  | 2 | 146,588 (19.4%) | 26,944 (21.4%) | 0.05 |
|  | 3 | 151,765 (20.0%) | 27,351 (21.7%) | 0.04 |
|  | 4 | 158,528 (20.9%) | 23,566 (18.7%) | 0.06 |
|  | 5 | 163,666 (21.6%) | 19,329 (15.3%) | 0.16 |
|  |  |  |  |  |
| LHIN (Local Health Integrated Network) | 1 ( Erie St. Clair) | 63,885 (8.4%) | 12,497 (9.9%) | 0.05 |
|  | 2 (South West) | 27,436 (3.6%) | 2,482 (2.0%) | 0.1 |
|  | 3 (Waterloo Wellington) | 64,100 (8.5%) | 7,661 (6.1%) | 0.09 |
|  | 4 (Hamilton Niagara Haldimand Brant) | 42,367 (5.6%) | 8,348 (6.6%) | 0.04 |
|  | 5 (Central West) | 44,979 (5.9%) | 18,231 (14.5%) | 0.28 |
|  | 6 (Mississauga Halton) | 86,463 (11.4%) | 20,390 (16.2%) | 0.14 |
|  | 7 (Toronto Central) | 68,652 (9.1%) | 7,887 (6.3%) | 0.11 |
|  | 8 (Central) | 87,317 (11.5%) | 21,391 (17.0%) | 0.16 |
|  | 9 (Central East) | 86,710 (11.5%) | 15,172 (12.0%) | 0.02 |
|  | 10 (South East) | 35,919 (4.7%) | 1,192 (0.9%) | 0.23 |
|  | 11 (Champlain) | 29,591 (3.9%) | 2,389 (1.9%) | 0.12 |
|  | 12 (North Simcoe Muskoka) | 49,801 (6.6%) | 4,178 (3.3%) | 0.15 |
|  | 13 (North East) | 39,521 (5.2%) | 1,968 (1.6%) | 0.2 |
|  | 14 (North West) | 30,398 (4.0%) | 2,230 (1.8%) | 0.13 |
|  |  |  |  |  |
| COVID-19 test result | Indeterminate | 1,043 (0.1%) | 0 (0.0%) | 0.05 |
|  | Negative | 756,096 (99.9%) | 0 (0.0%) | 38.08 |
|  | Positive | 0 (0.0%) | 126,016 (100.0%) | . |
|  |  |  |  |  |
| South Asians |  | 29,885 (3.9%) | 10,991 (8.7%) | 0.2 |
|  |  |  |  |  |
| Exposure | South Asian vaccinated | 11,859 (1.6%) | 422 (0.3%) | 0.13 |
|  | South Asian non vaccinated | 18,026 (2.4%) | 10,569 (8.4%) | 0.27 |
|  | non South Asian vaccinated | 254,587 (33.6%) | 7,575 (6.0%) | 0.74 |
|  | non South Asian non vaccinated | 472,667 (62.4%) | 107,450 (85.3%) | 0.54 |
|  |  |  |  |  |
| vaccination status | missing | 45,797 (6.0%) | 14,964 (11.9%) | 0.21 |
|  | 1 - Initiation | 11,034 (1.5%) | 2,652 (2.1%) | 0.05 |
|  | 2 - Complete | 209,995 (27.7%) | 52,460 (41.6%) | 0.3 |
|  | 3 - Complete with booster | 387,783 (51.2%) | 49,201 (39.0%) | 0.25 |
|  | 4 - Complete with 2 boosters | 102,530 (13.5%) | 6,739 (5.3%) | 0.28 |
|  |  |  |  |  |
| Completed vaccination until now |  | 700,308 (92.5%) | 108,400 (86.0%) | 0.21 |
|  |  |  |  |  |
