# Supplementary material for: COVID-19 vaccine effectiveness among South Asians in Canada
Source: PLOS Glob Public Health. 2024 Aug 1;4(8):e0003490. doi: 10.1371/journal.pgph.0003490 (PMC11293718; doi:10.1371/journal.pgph.0003490)
Supplement: S4 Table — (DOCX) [file pgph.0003490.s004.docx]

| **S4 Table : Baseline comorbidities of the overall cohort stratified by ethnicity and vaccination status** | | | | | |
| --- | --- | --- | --- | --- | --- |
| **Characteristics** | **South Asian vaccinated** | **South Asian non vaccinated** | **non-South Asian vaccinated** | **non-South Asian non vaccinated** | **P-VALUE** |
| Any comorbidity | 5,183 (42.2%) | 11,797 (41.3%) | 126,632 (48.3%) | 270,918 (46.7%) | <.001 |
| Respiratory conditions | | | | | |
| Chronic respiratory disease (Asthma, COPD) | 2,385 (19.4%) | 5,197 (18.2%) | 67,253 (25.7%) | 147,703 (25.5%) | <.001 |
| Asthma | 2,192 (17.8%) | 4,785 (16.7%) | 53,458 (20.4%) | 118,571 (20.4%) | <.001 |
| Chronic Obstructive Pulmonary Disease | 359 (2.9%) | 769 (2.7%) | 21,439 (8.2%) | 45,075 (7.8%) | <.001 |
| Cardiometabolic conditions | | | | | |
| Chronic heart disease (congestive heart failure, ischemic heart disease, atrial fibrillation) | 631 (5.1%) | 1,326 (4.6%) | 21,150 (8.1%) | 40,039 (6.9%) | <.001 |
| Heart failure | 204 (1.7%) | 450 (1.6%) | 8,223 (3.1%) | 15,429 (2.7%) | <.001 |
| Ischemic heart disease | 323 (2.6%) | 695 (2.4%) | 8,117 (3.1%) | 15,924 (2.7%) | <.001 |
| Atrial fibrillation | 303 (2.5%) | 590 (2.1%) | 12,450 (4.7%) | 22,776 (3.9%) | <.001 |
| Angina | 36 (0.3%) | 79 (0.3%) | 888 (0.3%) | 1,831 (0.3%) | 0.159 |
| Chronic ischemic heart disease | 173 (1.4%) | 356 (1.2%) | 4,272 (1.6%) | 8,437 (1.5%) | <.001 |
| Myocardial infarction | 71 (0.6%) | 190 (0.7%) | 2,369 (0.9%) | 4,872 (0.8%) | <.001 |
| Coronary artery bypass graft | 94 (0.8%) | 189 (0.7%) | 2,179 (0.8%) | 4,116 (0.7%) | <.001 |
| Percutaneous coronary intervention | 224 (1.8%) | 473 (1.7%) | 4,747 (1.8%) | 9,353 (1.6%) | <.001 |
| Hypertension | 2,390 (19.5%) | 5,649 (19.8%) | 59,883 (22.8%) | 120,770 (20.8%) | <.001 |
| Diabetes | 1,889 (15.4%) | 4,471 (15.6%) | 29,797 (11.4%) | 62,735 (10.8%) | <.001 |
| Other comorbid conditions | | | | | |
| Immunocompromised disorders | 367 (3.0%) | 790 (2.8%) | 14,279 (5.4%) | 28,669 (4.9%) | <.001 |
| HIV | 12 (0.1%) | 22 (0.1%) | 489 (0.2%) | 1,084 (0.2%) | <.001 |
| Solid organ transplant or bone marrow transplant | 27 (0.2%) | 53 (0.2%) | 936 (0.4%) | 1,369 (0.2%) | <.001 |
| Sickle cell anemia | <=5 (0.0%) | <=5 (0.0%) | 59 (0.0%) | 219 (0.0%) | <.001 |
| Other immune system disorders | 127 (1.0%) | 287 (1.0%) | 4,840 (1.8%) | 9,855 (1.7%) | <.001 |
| immunosuppressive therapy | 138 (1.1%) | 290 (1.0%) | 5,404 (2.1%) | 9,774 (1.7%) | <.001 |
| Treatment for cancer in past 6m or recent diagnosis | 102 (0.8%) | 229 (0.8%) | 4,656 (1.8%) | 10,162 (1.8%) | <.001 |
| Organ transplant | 17 (0.1%) | 39 (0.1%) | 569 (0.2%) | 820 (0.1%) | <.001 |
| Bone marrow/stem cell transplant | 10 (0.1%) | 14 (0.0%) | 370 (0.1%) | 555 (0.1%) | <.001 |
| Autoimmune disease | 433 (3.5%) | 891 (3.1%) | 11,841 (4.5%) | 23,870 (4.1%) | <.001 |
| Rheumatoid arthritis | 152 (1.2%) | 322 (1.1%) | 3,602 (1.4%) | 7,190 (1.2%) | <.001 |
| Inflammatory bowel disease | 73 (0.6%) | 143 (0.5%) | 1,904 (0.7%) | 3,874 (0.7%) | <.001 |
| Psoriasis | 212 (1.7%) | 402 (1.4%) | 5,508 (2.1%) | 11,079 (1.9%) | <.001 |
| Psoriatic arthritis | 24 (0.2%) | 38 (0.1%) | 674 (0.3%) | 1,270 (0.2%) | <.001 |
| Multiple sclerosis | 15 (0.1%) | 51 (0.2%) | 1,193 (0.5%) | 2,501 (0.4%) | <.001 |
| Chronic kidney disease or dialysis | 354 (2.9%) | 723 (2.5%) | 9,202 (3.5%) | 16,975 (2.9%) | <.001 |
| Chronic kidney disease | 354 (2.9%) | 723 (2.5%) | 9,202 (3.5%) | 16,973 (2.9%) | <.001 |
| Dialysis in each of 3 months prior to index date | 16 (0.1%) | 46 (0.2%) | 642 (0.2%) | 1,080 (0.2%) | <.001 |
| Advanced liver disease | 84 (0.7%) | 212 (0.7%) | 2,733 (1.0%) | 6,055 (1.0%) | <.001 |
| Cirrhosis | 79 (0.6%) | 201 (0.7%) | 2,565 (1.0%) | 5,714 (1.0%) | <.001 |
| Decompensated cirrhosis | 9 (0.1%) | 31 (0.1%) | 431 (0.2%) | 972 (0.2%) | 0.007 |
| Dementia | 93 (0.8%) | 164 (0.6%) | 4,868 (1.9%) | 7,798 (1.3%) | <.001 |
| Transient ischemic attack or stroke | 90 (0.7%) | 193 (0.7%) | 4,193 (1.6%) | 7,832 (1.4%) | <.001 |
| Transient ischemic attack | 50 (0.4%) | 111 (0.4%) | 2,640 (1.0%) | 4,775 (0.8%) | <.001 |
| Acute ischemic stroke | 45 (0.4%) | 97 (0.3%) | 1,956 (0.7%) | 3,738 (0.6%) | <.001 |
