# Supplementary material for: COVID-19 vaccine effectiveness among South Asians in Canada
Source: PLOS Glob Public Health. 2024 Aug 1;4(8):e0003490. doi: 10.1371/journal.pgph.0003490 (PMC11293718; doi:10.1371/journal.pgph.0003490)
Supplement: S6 Table — (DOCX) [file pgph.0003490.s006.docx]

**S6 Table:** Vaccine effectiveness among South Asians and non-South Asians when COVID-19 associated hospitalization and deaths were analysed separately

| **Outcome** | **Effect** | **Odds Ratio** | **Lower CI** | **Upper CI** | **Vaccine effectiveness** | **Vaccine effectiveness lower CI** | **Vaccine effectiveness upper CI** |
| --- | --- | --- | --- | --- | --- | --- | --- |
| Hospitalization associated with symptomatic COVID-19 infection | South Asian vaccinated vs South Asian non-vaccinated  (n=30411) | 0.02 | 0.01 | 0.05 | 97.7 | 95.4 | 98.9 |
|  | non-South-Asian vaccinated vs non-South-Asian non-vaccinated  (n=734742) | 0.06 | 0.06 | 0.07 | 93.6 | 92.8 | 94.4 |
| Death associated with symptomatic COVID-19 infection | South Asian vaccinated vs South Asian non-vaccinated  (n=29947) | 0.05 | 0.01 | 0.19 | 95.5 | 81.5 | 98.9 |
|  | non-South-Asian vaccinated vs non-South-Asian non-vaccinated  (n=728503) | 0.10 | 0.08 | 0.13 | 89.8 | 87.2 | 91.9 |
