# Supplementary material for: COVID-19 vaccine effectiveness among South Asians in Canada
Source: PLOS Glob Public Health. 2024 Aug 1;4(8):e0003490. doi: 10.1371/journal.pgph.0003490 (PMC11293718; doi:10.1371/journal.pgph.0003490)
Supplement: S7 Table — (DOCX) [file pgph.0003490.s007.docx]

**S7 Table:** Vaccine effectiveness among South Asians and non-South Asians after excluding those with pre-existing respiratory conditions.

| **Outcome** | **Effect** | **Odds Ratio** | **Lower CI** | **Upper CI** | **Vaccine effectiveness** | **Vaccine effectiveness lower CI** | **Vaccine effectiveness upper CI** |
| --- | --- | --- | --- | --- | --- | --- | --- |
| Symptomatic covid19 infection | South Asian vaccinated vs South Asian non-vaccinated  (n=33294) | 0.062 | 0.055 | 0.069 | 93.8 | 93.1 | 94.5 |
|  | non-South-Asian vaccinated vs non-South-Asian non-vaccinated  (n=627323) | 0.128 | 0.125 | 0.132 | 87.2 | 86.8 | 87.5 |
| Hospitalization or  death associated with symptomatic COVID-19 infection | South Asian vaccinated vs South Asian non-vaccinated  (n=24584) | 0.028 | 0.014 | 0.056 | 97.2 | 94.4 | 98.6 |
|  | non-South-Asian vaccinated vs non-South-Asian non-vaccinated  (n=542239) | 0.055 | 0.047 | 0.063 | 94.5 | 93.7 | 95.3 |
