# Supplementary material for: COVID-19 vaccine effectiveness among South Asians in Canada
Source: PLOS Glob Public Health. 2024 Aug 1;4(8):e0003490. doi: 10.1371/journal.pgph.0003490 (PMC11293718; doi:10.1371/journal.pgph.0003490)
Supplement: S10 Table — (DOCX) [file pgph.0003490.s010.docx]

**S10 Table:** Adjusted logistic regression models for outcomes of COVID-19 related hospitalizations and death analyzed separately in non-vaccinated South Asians, stratified by immigration status and reason for immigration (Referent cohort: non-South Asian non-vaccinated)

| **Overall cohort** | | **Non-immigrants** | | **Recent immigrant (<10 years)** | | **Non-recent immigrant (>10 years)** | |
| --- | --- | --- | --- | --- | --- | --- | --- |
| Covid-19 related hospitalization (n=765153) | Covid-19 related death (n=758450) | Covid-19 related hospitalization (n=635122) | Covid-19 related death (n=630943) | Covid-19 related hospitalization (n=38035) | Covid-19 related death (n=37512) | Covid-19 related hospitalization (n=91996) | Covid-19 related death (n=89995) |
| 2.05  (1.9, 2.2) | 1.90  (1.5, 2.5) | 2.3  (2.0, 2.6) | 2.4  (1.6, 3.4) | 0.85  (0.6, 1.1) | 0.5  (0.1, 2.2) | 1.02  (0.9, 1.2) | 0.8  (0.6, 1.2) |
| **Total immigrants** | | **Economic** | | **Refugee** | | **Family/others** | |
| Covid-19 related hospitalization (n=130031) | Covid-19 related death  (n=127507) | Covid-19 related hospitalization (n=68443) | Covid-19 related death  (n=67531) | Covid-19 related hospitalization (n=19304) | Covid-19 related death  (n=18709) | Covid-19 related hospitalization (n= 42284) | Covid-19 related death (n=41267) |
| 0.98  (0.9, 1.1) | 0.80  (0.5, 1.2) | 1.0  (0.8, 1.2) | 0.9  (0.4, 2.1) | 0.6  (0.4, 0.9) | 0.8  (0.3, 2.2) | 1.2  (1.0, 1.4) | 0.8  (0.5, 1.3) |
