# Supplementary material for: COVID-19 vaccine effectiveness among South Asians in Canada
Source: PLOS Glob Public Health. 2024 Aug 1;4(8):e0003490. doi: 10.1371/journal.pgph.0003490 (PMC11293718; doi:10.1371/journal.pgph.0003490)
Supplement: S11 Table — (DOCX) [file pgph.0003490.s011.docx]

**S11 Table:** Adjusted logistic regression models for various outcomes of COVID-19 in non-vaccinated South Asians, **stratified by immigration status and reason for immigration** (Referent cohort: non-South Asian non-vaccinated) after excluding those with pre-existing respiratory conditions

| **Overall cohort** | | **Non-immigrants** | | **Recent immigrant (<10 years)** | | **Non-recent immigrant (>10 years)** | |
| --- | --- | --- | --- | --- | --- | --- | --- |
| Covid-19 related hospitalization or death (n=566823) | Covid-19 infection (n=660617) | Covid-19 related hospitalization or death (n=453260) | Covid-19 infection (n=510735) | Covid-19 related hospitalization or death (n=36414) | Covid-19 infection (n=48843) | Covid-19 related hospitalization or death (n=77149) | Covid-19 infection (n=101039) |
| 1.84  (1.67, 2.04) | 2.45  (2.18, 2.31) | 2.2  (1.8, 2.6) | 2.5  (2.4, 2.6) | 0.8  (0.6, 1.4) | 1.2  (1.1, 1.2) | 1.0  (0.8, 1.1) | 1.4  (1.3, 1.4) |
| **Total immigrants** | | **Economic** | | **Refugee** | | **Family/others** | |
| Covid-19 related hospitalization or death (n=113563) | Covid-19 infection (n=149882) | Covid-19 related hospitalization or death (n=60688) | Covid-19 infection  (n=75276) | Covid-19 related hospitalization or death (n=16326) | Covid-19 infection  (n=23920) | Covid-19 related hospitalization or death (n= 36549) | Covid-19 infection (n=50686) |
| 0.93  (0.82, 1.01) | 1.29  (1.25, 1.34) | 1.0  (0.8, 1.2) | 1.3  (1.2, 1.3) | 0.7  (0.5, 1.0) | 1.1  (1.5, 1.6) | 1.0  (0.9, 1.2) | 1.5  (1.5, 1.6) |
