# Supplementary material for: COVID-19 vaccine effectiveness among South Asians in Canada
Source: PLOS Glob Public Health. 2024 Aug 1;4(8):e0003490. doi: 10.1371/journal.pgph.0003490 (PMC11293718; doi:10.1371/journal.pgph.0003490)
Supplement: S12 Table — (DOCX) [file pgph.0003490.s012.docx]

**S12 Table: Vaccine effectiveness among South Asians and non-South Asians in Wave 2 of COVID-19 pandemic [(**Dec 14 2020 (start of our data) to 28 February 2021]

| **Outcome** | **Effect** | **Odds Ratio** | **Lower CI** | **Upper CI** | **Vaccine effectiveness** | **Vaccine effectiveness lower CI** | **Vaccine effectiveness upper CI** |
| --- | --- | --- | --- | --- | --- | --- | --- |
| Symptomatic COVID-19 infection | South Asian vaccinated vs South Asian non-vaccinated  N= 8627 | 0.104 | 0.042 | 0.256 | 89.6 | 74.4 | 95.8 |
|  | non-South-Asian vaccinated vs non-South-Asian non-vaccinated  n= 21296 | 0.136 | 0.109 | 0.171 | 86.4 | 82.3 | 89.1 |
| Hospitalization or  death associated with symptomatic COVID-19 infection | South Asian vaccinated vs South Asian non-vaccinated  N= 5798 | <0.001 | <0.001 | >999.99 | 99.9 | -- | 100 |
|  | non-South-Asian vaccinated vs non-South-Asian non-vaccinated  n= 181567 | 0.065 | 0.021 | 0.203 | 93.5 | 79.7 | 97.9 |
