# Supplementary material for: COVID-19 vaccine effectiveness among South Asians in Canada
Source: PLOS Glob Public Health. 2024 Aug 1;4(8):e0003490. doi: 10.1371/journal.pgph.0003490 (PMC11293718; doi:10.1371/journal.pgph.0003490)
Supplement: S13 Table — (DOCX) [file pgph.0003490.s013.docx]

**S13 Table: Vaccine effectiveness among South Asians and non-South Asians in Wave 3 of COVID-19 pandemic (**March 1 2021 to July 31 2021)

| **Outcome** | **Effect** | **Odds Ratio** | **Lower CI** | **Upper CI** | **Vaccine effectiveness** | **Vaccine effectiveness lower CI** | **Vaccine effectiveness upper CI** |
| --- | --- | --- | --- | --- | --- | --- | --- |
| Symptomatic COVID-19 infection | South Asian vaccinated vs South Asian non-vaccinated  N= 20627 | 0.056 | 0.043 | 0.073 | 94.4 | 92.7 | 95.7 |
|  | non-South-Asian vaccinated vs non-South-Asian non-vaccinated  n= 368820 | 0.103 | 0.097 | 0.11 | 89.7 | 89.1 | 90.3 |
| Hospitalization or  death associated with symptomatic COVID-19 infection | South Asian vaccinated vs South Asian non-vaccinated  N= 13590 | 0.085 | 0.038 | 0.191 | 91.5 | 80.9 | 96.2 |
|  | non-South-Asian vaccinated vs non-South-Asian non-vaccinated  n= 308713 | 0.074 | 0.06 | 0.091 | 92.6 | 90.9 | 94.1 |
