# Supplementary material for: COVID-19 vaccine effectiveness among South Asians in Canada
Source: PLOS Glob Public Health. 2024 Aug 1;4(8):e0003490. doi: 10.1371/journal.pgph.0003490 (PMC11293718; doi:10.1371/journal.pgph.0003490)
Supplement: S14 Table — (DOCX) [file pgph.0003490.s014.docx]

**S14 Table: Vaccine effectiveness among South Asians and non-South Asians in Wave 4 of COVID-19 pandemic (**Wave 4: August 1 2021 to November 15, 2021 (end of our data).

| **Outcome** | **Effect** | **Odds Ratio** | **Lower CI** | **Upper CI** | **Vaccine effectiveness** | **Vaccine effectiveness lower CI** | **Vaccine effectiveness upper CI** |
| --- | --- | --- | --- | --- | --- | --- | --- |
| Symptomatic COVID-19 infection | South Asian vaccinated vs South Asian non-vaccinated  N= 11622 | 0.163 | 0.135 | 0.198 | 83.6 | 80.2 | 86.5 |
|  | non-South-Asian vaccinated vs non-South-Asian non-vaccinated  n= 260663 | 0.113 | 0.109 | 0.117 | 88.7 | 88.3 | 89.1 |
| Hospitalization or  death associated with symptomatic COVID-19 infection | South Asian vaccinated vs South Asian non-vaccinated  N= 11085 | 0.021 | 0.006 | 0.066 | 97.9 | 93.4 | 99.34 |
|  | non-South-Asian vaccinated vs non-South-Asian non-vaccinated  n= 245621 | 0.03 | 0.026 | 0.034 | 97.1 | 96.6 | 97.4 |
