# Supplementary material for: COVID-19 vaccine effectiveness among South Asians in Canada
Source: PLOS Glob Public Health. 2024 Aug 1;4(8):e0003490. doi: 10.1371/journal.pgph.0003490 (PMC11293718; doi:10.1371/journal.pgph.0003490)
Supplement: S1 Fig — (DOCX) [file pgph.0003490.s015.docx]

**S1 Figure: Participant flow diagram**

**Cases**

Positive covid-19 tests from 2020-12-14 to 2021-11-15 and symptoms

(n=153,927 )

**Controls**

Negative covid-19 tests from 2020-12-14 to 2021-11-15 and symptoms

N=1,096,777

**Total cases and controls**

N= 1,250,704

**Excluded individuals**

- Age < 18 and not residing in Ontario, n= 344,411

- Living in long term care (LTC), n= 19,968

- Positive covid-19 test before 2020-12-13, n= 799

- Not eligible for health care coverage at index date, n= 2371

**Final Cohort**

N= 883,155

(Cases: 126,016)

(Controls: 757,139)

South Asian vaccinated

N= 12,881

South Asian non-vaccinated

N= 28,595

Non-South Asian vaccinated

N= 262,162

Non-South Asian non-vaccinated

N= 580,117
