# Supplementary material for: COVID-19 vaccine effectiveness among South Asians in Canada
Source: PLOS Glob Public Health. 2024 Aug 1;4(8):e0003490. doi: 10.1371/journal.pgph.0003490 (PMC11293718; doi:10.1371/journal.pgph.0003490)
Supplement: Attachment — Submitted filename: response to reviewers.docx [file pgph.0003490.s016.docx]

We thank the Editors and Reviewers for their thoughtful feedback.

As the revisions required additional analysis and our statistician, Jin Luo was on vacation, we had to ask Dr. Karl Everett to perform this analysis. For that reason, we have added him as a co-author in this revised version.

Review Comments to the Author

**Reviewer #1: I do have some comments/feedback:**

**Line 61-62: Was there an IRB non-research determination?**

**Comment:** The Ontario Personal Health Information Protection Act authorized the use of data for this study without review by a Research Ethics Board. We have edited the paragraph (lines 58-60).

**Line 78: Why one at random in determining the index and not the first one? Why not the CSTE case definition as is used in the literature. DO you have a justification for using this? If not, I recommend using the CSTE case definition including the 90 day rule for reinfections found in a the following site.**
<https://ndc.services.cdc.gov/case-definitions/coronavirus-disease-2019-2021/>

**Comment**: We thank the reviewer for this question. Selecting a random test introduces less bias than choosing the first, since people are less likely to be vaccinated at earlier times.

Regarding choosing the specific case definition, we used the definition based on what data were collected at the time of SARS CoV2 testing in Ontario and followed a similar strategy as published in the BMJ earlier using these databases (<https://www.bmj.com/content/374/bmj.n1943>).

**Lines 82-83: Why not the CDC recommended definition for fully vaccinated which is 14 days after the 2nd dose? Do you have a justification for this? If not, I recommend using the CDC recommended definition used in the literature. At a minimum please add a sensitivity analysis using the 14 day definition.**

**Comment:** Covid-19 vaccine effectiveness is generally defined 14 days after the first dose but 7 days after the 2^nd^ dose. We only included individuals with 2 doses in our study, so our timing started 7 days after the 2^nd^ dose as suggested by this NEJM paper (<https://www.nejm.org/doi/10.1056/NEJMoa2101765>)

and the BMJ paper (<https://www.bmj.com/content/374/bmj.n1943>).

**Lines 104-105: This loo(1)ks like the CSTE definition for hospitalizations. I suggest referencing that.**

**Comment:** We have included the reference (Line 110)

**Tables 1 and 2: Is this p value for a group chi-square test? IF so can you specify?**

**Comment:** Yes, we have added this to the analysis section (Line 127)

Comments on statistical analysis:
**I suggest adding overall model goodness of fit statistics associated with tables 3 and 4.
Close to 30% of the total cases and controls we excluded due to various reasons including due to missing values. Not sure what % of that is <18 years. For others, recommend conducting a sensitivity analysis or at a minimum discuss the potential impact/limitations.**

**Comment:** We thank the reviewer for this question. Of 344,411 participants excluded, 342,409 (99.4%) were <18 years of age. We have included the Hosmer-Lemeshow test p-value as the model goodness in the tables as suggested.

**Comment on data availability:
Whether the files made available is the matched analysis file or the original data is not clear. If possible, suggest making the matched analysis file available.**

Comments: The dataset from this study is held securely in coded form at ICES. Legal data sharing agreements between ICES and data providers (e.g., healthcare organizations and government) prohibit ICES from making the dataset publicly available. We have further clarified this in the manuscript. (Lines 290-93)

**Reviewer #2:** This study evaluates the effectiveness of COVID-19 vaccines among South Asians compared to non-South Asians in Ontario, Canada, using a test-negative study design. It reveals a high COVID-19 vaccine effectiveness among South Asians in Ontario and indicates that non-vaccinated South Asians have higher odds of symptomatic COVID-19 infection. Overall, the study is well-written and informative, but I have several suggestions, particularly regarding the statistical methods employed, which I believe could enhance the paper:

**• Have hospitalizations and deaths been investigated separately? I could imagine that hospitalizations drive a bulk of the results and would be a relevant sensitivity analysis.**

**Comment:** We have performed a sensitivity analysis to analyse hospitalizations and deaths associated with COVID-19 separately. We have modified the results section and added those tables in the supplementary section.

**• Line 66: Clarification is needed on how symptoms consistent with COVID-19 are defined. Did you use certain diagnostic codes or is it based on a PCR test?**

**Comment:** Information on symptoms were collected from patients at the time of testing and are recorded in the Ontario Laboratory Information System alongside the PCR test results. We have added this to the manuscript. (Lines 94-95)

**• Table 2 & Line 123: Are all comorbidities listed in Table 2 combined in the model as one covariate for any comorbidity? If so, consider incorporating a score or grouping comorbidities (e.g., respiratory, cardiovascular) for a more comprehensive analysis. Having an extensive Table 2 with all the comorbidities represented and then not being as detailed with the covariate in the analysis seems excessive. Moving Table 2 to the supplementary section and presenting the score in Table 1 could improve clarity. Additionally, conducting sensitivity analyses, such as excluding individuals with preexisting respiratory conditions or stratifying by them, could be informative. One could hypothesize that having preexisting conditions related to COVID could make you more susceptible.**

**Comment**:

We agree with the reviewer that currently all comorbidities are combined in the model as one covariate. Based on the suggestion, we have now regrouped them into respiratory, cardiac, cardiometabolic and other and moved this table to supplementary section.

We have also performed sensitivity analysis after excluding those with pre-existing respiratory conditions and have modified methods/results section and also included those tables in the supplemental section.

**• Line 128: Missing hyphen in non-vaccinated.**

**Comment:** We have added a hyphen

**• Line 136: Including 126,016 and 757,139 in the flowchart for completeness would enhance clarity.**

**Comment:** We have edited the flow diagram accordingly.

**• Lines 145-146: Immigrant percentages mentioned are not found in any of the tables.**

Comment: We have added those numbers in Table 1

**• Table 4: I find table 4 confusing, especially the fact that it has two ‘Overall’ categories. I would recommend having more informative titles.**

**Comment:** We have changed the categories in table 4.

**• Logistic regression: The use of logistic regression may overlook the time-varying nature of the COVID-19 pandemic and assumes a static relationship between predictors and outcomes. Given the influence of temporal factors such as COVID infections, variants, seasonal variations, and vaccination roll-outs, Cox regression or conditional logistic regression, would be a more suitable approach. The conditional logistic regression could potentially be matched on time. VE could be confounded by changes in the underlying risk of COVID-19 infection over time, leading to biased results. An initial check to see if there is any time-varying confounding is to conduct a sensitivity analysis to see if stratifying by time periods has an effect on your estimates.**

**Comment:** We didn’t use time varying confounders as this is a nested case control study which allows only logistic regression model to be applied for studying the outcomes.

• **Given the variant variation and the fluctuating effectiveness of vaccines dependent on variants, could you provide a short description of the variant dominance in Canada during the study period? The timing of vaccination could also have an effect on the effectiveness in this year-long follow-up. Consider including this covariate as an adjuster in your analysis.**

**Comment:** The important variants during our study period were Alpha (B.1.1.7), Beta (B.1.351), Gamma (P.1), Delta (B.1.617.2), Omicron (B.1.1.529). (<https://health-infobase.canada.ca/covid-19/archive/2022-06-10/#VOC>). We have added this in the manuscript (lines 153-54).

We thank the reviewer for this question about adjusting for the timing of vaccination. However, our study period ended Nov 2021. Therefore, none of the participants could be >1 year since their vaccination. Hence, examining timing of vaccination is not feasible with our current study, but should be considered in future studies with longer follow-up times.
