# Supplementary material for: COVID-19 vaccine effectiveness among South Asians in Canada
Source: PLOS Glob Public Health. 2024 Aug 1;4(8):e0003490. doi: 10.1371/journal.pgph.0003490 (PMC11293718; doi:10.1371/journal.pgph.0003490)
Supplement: Attachment — Submitted filename: response to reviewers_v3.docx [file pgph.0003490.s017.docx]

We thank the Editors and Reviewers for their thoughtful feedback.

Journal Requirements:

1. Please amend your detailed online Financial Disclosure statement. This is published with the article. It must therefore be completed in full sentences and contain the exact wording you wish to be published.

a) State the initials, alongside each funding source, of each author to receive each grant, if applicable. For example: "This work was supported by the National Institutes of Health (####### to AM; ###### to CJ) and the National Science Foundation (###### to AM)."
b) State what role the funders took in the study. If the funders had no role in your study, please state: “The funders had no role in study design, data collection and analysis, decision to publish, or preparation of the manuscript.”

**Comment:** This study was supported by ICES, which is funded by an annual grant from the Ontario Ministry of Health and Long-Term Care, the COVID-19 Immunity Task Force, Public Health Agency of Canada (2122-HQ-000058 to SSA), and the Ontario Health Data Platform, a Province of Ontario initiative to support Ontario’s ongoing response to COVID-19 and its related impacts. Sonia Anand holds a Tier 1 Canada Research Chair in Ethnic Diversity and Cardiovascular Disease (#CRC-2017-00024 to SSA), and Michael G. DeGroote Heart and Stroke Foundation Chair in Population Health. The funders had no role in study design, data collection and analysis, decision to publish, or preparation of the manuscript.

2. Please ensure that the funders and grant numbers match between the Financial Disclosure field and the Funding Information tab in your submission form. Note that the funders must be provided in the same order in both places as well.

Comment: We have included grant numbers in the submission form online

3. Please review your reference list to ensure that it is complete and correct. If you have cited papers that have been retracted, please include the rationale for doing so in the manuscript text, or remove these references and replace them with relevant current references. Any changes to the reference list should be mentioned in the rebuttal letter that accompanies your revised manuscript. If you need to cite a retracted article, indicate the article’s retracted status in the References list and also include a citation and full reference for the retraction notice.

**Comment:** We have verified the reference list

Reviewers' comments:

Reviewer's Responses to Questions

**Comments to the Author**

Please use the space provided to explain your answers to the questions above. You may also include additional comments for the author, including concerns about dual publication, research ethics, or publication ethics. (Please upload your review as an attachment if it exceeds 20,000 characters)

Reviewer #1: na

Reviewer #2: Line 119: Please specify the 4 groups

**Comment:** We have specified the 4 groups

Line 139: “A total of 883,155 individuals who had PCR test” -> “A total of 883,155 individuals who had a PCR test”

Comment: We have edited it accordingly

Table 3: The footnotes in table 3 look like exponents. I would recommend changing this notation. You could just include the p-value output in the table and add row titles or add the footnote to the subtitle.

**Comment:** We have modified the table to comply with journal requirements

Line 200: isn’t the 2nd and 4th wave also included in the study period?

**Comment:** We have modified the statement and have included 2^nd^ and 4^th^ waves

I still think there is a potential issue with temporal timing that is not addressed. During the study period, COVID came in separate waves – some perhaps more severe than others. If most individuals get vaccinated towards the middle/end of the study, then the individuals who are in the vaccinated group most likely only consist of the last wave in the study period. Furthermore, the unvaccinated would then span over the whole period while the vaccinated most likely includes those who test positive towards the end of the period, giving an uneven opportunity for a positive test throughout the study, potentially biasing the result towards more effect. As a sensitivity analysis, this could be checked by stratifying the analysis by the study period (tests before/after June, for example), or otherwise limiting your study period to a time when most individuals have been vaccinated (e.g., > June). By limiting it to a time period where vaccination coverage is more uniform, you can check if the effect of vaccination is constant over time periods. Alternatively, the methods paper you refer to (15) stratifies by the following to take into account the effect of waves and variants: “epidemic wave (index dates 14 December 2020 to 7 February 2021, representing wave 2 in Ontario; 8 February 2021 to 21 March 2021, representing the period between wave 2 and wave 3; and 22 March 2021 to 19 April 2021, representing wave 3), and variant (earlier variant versus alpha versus beta or gamma).”

**Comment:** We thank the reviewer for this comment. We have now added a stratified analysis by the pandemic waves and found results similar to the main analysis (supplementary tables 12-14)
